# Clinical and Molecular Risk Factors for Recurrence Following Radical Surgery of Well-Differentiated Pancreatic Neuroendocrine Tumors

**DOI:** 10.3389/fmed.2020.00385

**Published:** 2020-08-05

**Authors:** Alessandra Pulvirenti, Antonio Pea, David K. Chang, Nigel B. Jamieson

**Affiliations:** ^1^Unit of General and Pancreatic Surgery, University and Hospital Trust of Verona, Verona, Italy; ^2^Wolfson Wohl Cancer Research Centre, Institute of Cancer Sciences, University of Glasgow, Glasgow, United Kingdom; ^3^West of Scotland Pancreatic Unit, Glasgow Royal Infirmary, Glasgow, United Kingdom

**Keywords:** pancreatic neuroendocrine tumors, neuroendocrine tumors, pancreatic surgery, recurrence, molecular markers

## Abstract

Well-differentiated pancreatic neuroendocrine tumors are increasingly diagnosed neoplasms. For localized disease, surgery is the first-line therapy and is curative in most cases. However, although recurrence is a rare event, it can still occur up to 10 years from surgery, worsening the prognosis. Many clinical and pathological factors have been associated with recurrence; however, it is currently unclear how to accurately discern patients at risk for relapse of disease from those that should be considered cured. In this review, we focus on clinical, pathological, and molecular factors associated with recurrence and discuss available prediction tools to assess the risk of recurrence following surgery.

## Introduction

Pancreatic neuroendocrine tumor (PanNET) is a heterogeneous group of neoplasms expressing hormones and general markers of neuroendocrine differentiation (Table 1) (1). Once considered rare tumors, the incidence of PanNETs has increased significantly over the last decades. Data from the US SEER database have shown that the number of new diagnoses per year rose almost 3-fold from 2000 to 2012, reaching 0.8 cases per 100,000 individuals (2). The increase of diagnoses has mostly concerned asymptomatic patients with localized low-/intermediate-grade tumors, due to the widespread use of cross-sectional imaging modalities. As a consequence, the number of pancreatic resections for PanNET has risen; consequently, PanNET is the second most frequent indication for pancreatic surgery following pancreatic adenocarcinoma (3). In 2019, the World Health Organization (WHO) has reclassified pancreatic neuroendocrine neoplasms (PanNEN) to distinguish well-differentiated neuroendocrine tumors (PanNETs), including high-grade, from poorly differentiated carcinomas (PanNECs) (1). PanNECs are characterized by a different pathological cellular morphology, higher proliferative index, and molecular alterations that correspond to a dismal prognosis therefore clearly categorizing them from well-differentiated PanNETs (1, 4, 5). While surgical resection represents the first-line treatment for localized PanNETs and is curative in 70–90% of cases, it is not indicated for PanNECs due to their poor prognosis, with systemic chemotherapy generally preferred (4, 6–8). For patients with a PanNET undergoing surgical resection, the risk of recurrence is widely heterogeneous and can persist for up to 10 years. Conventional staging and grading systems have been used to risk stratify patients; however, these approaches consider only a limited number of variables and include patients with variable tumor biology and subsequently risk of recurrence can be misclassified (9–11). Over recent years, to improve prognostication and establish more personalized surveillance schedules, several nomograms and predictive risk models incorporating multiple variables have been developed.

**Table 1:** Pancreatic neuroendocrine neoplasm classification according to functional status and WHO classification (1).

**Functional status**
• Non-functioning^*^ • Functioning ▪ Insulinoma ▪ Gastrinoma ▪ Glucagonoma ▪ VIPoma ▪ Other (producing serotonin, ACTH, GHRH, PTHrp, and CCK)
**WHO classification**
• Well-differentiated pancreatic neuroendocrine tumor (PanNET) ▪ Grade 1 (low), Ki67 <3% ▪ Grade 2 (intermediate), Ki67 3–20% ▪ Grade 3 (high), Ki67 >20% • Poorly differentiated pancreatic neuroendocrine carcinoma (PanNEC), high-grade, Ki67 >20%

* *Non-functioning tumors may secrete hormones but are not associated with a clinical hormone hypersecretion syndrome*.

At the same time, the genomic landscape of PanNETs has been comprehensively characterized, reaffirming molecular alterations in telomere maintenance and the mTOR pathway as indicators of aggressive tumor behavior. In particular, functional silencing of *DAXX* or *ATRX* genes promote the activation of the alternative lengthening of telomere (ALT) pathway and are commonly associated with the development of distant metastases, while the clinical significance of other molecular alterations is currently debated.

To date, consensus is lacking on which patients should be enrolled in postoperative surveillance programs, on the frequency and the length of the follow-up period, and on the optimal imaging modalities to employ (10–13). Without accurate stratification of the risk of recurrence, many patients will potentially be exposed to unnecessary imaging studies over a protracted period.

The purpose of this review article is to summarize the current evidence on the predictive clinicopathological and risk factors for PanNET recurrence, including an overview of the clinical available predictive models to manage surveillance following surgery. Herein, we will discuss the existing molecular data and determine strategies to integrate these data into the current clinical practice to better predict recurrence.

## Recurrence After Curative Surgery

PanNET recurrence following curative surgery occurs in 8–17% of patients (9, 14, 15), significantly worsening the prognosis (14, 16). Data on patterns of recurrence are few and heterogeneous, due to several factors including the misclassification of high-grade PanNETs with PanNECs, the inconsistent inclusion of patients with a familial syndrome, and the heterogeneity of imaging protocols for diagnosis and follow-up of PanNET patients across countries and institutions.

**Figure 1:**
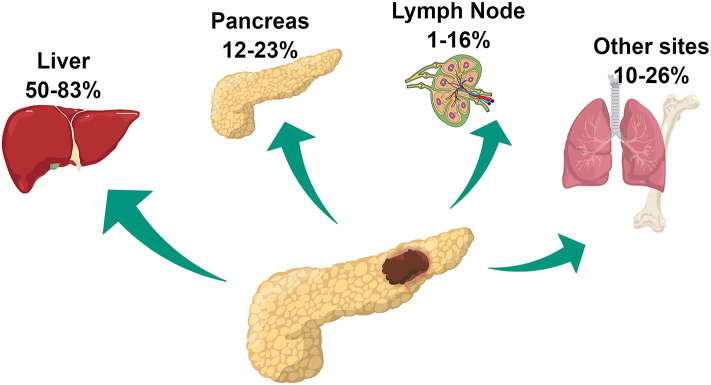
PanNET pattern of recurrence following surgical resection (14, 17).

Among patients undergoing surgery, commonly reported sites of recurrence are the liver, pancreas remnant, and lymph nodes (14, 17). Less frequently, other sites including lungs, bone, kidney, and peritoneum are involved (Figure 1). Liver involvement is the most frequent accounting for 50–83% of cases of recurrence. Data on the rate of pancreatic local recurrence and lymph nodes remains heterogeneous, ranging widely among surgical series, from 12–23% to 1–16%, respectively (14, 16, 17). While liver recurrence is associated with biological characteristics of the tumor and more specifically with a more aggressive phenotype, pancreatic local recurrence seems to be related to the presence of microscopical residual disease left on the surgical margins and therefore related to surgical procedure (14, 16). The discrepant rates of lymph nodal recurrence could be explained by the different imaging strategies employed during follow-up. The use of 68Ga-DOTATATE PET/CT has been approved in the USA by the Food and Drug Administration only in 2016, whereas its use for PanNET management had already been consolidated in Europe for several years. This imaging modality provides improved accuracy in identifying the presence of neuroendocrine disease compared to conventional imaging including Octreoscan and might have contributed to the higher rate of lymph-node recurrence reported in the European surgical series (18).

An important question concerns whether the risk of recurrence is decreasing over time. Retrospective studies (9, 14) report a median time to recurrence of 35–37 months from surgery, but several cases recurred up to 10 years, advocating long follow-up (9, 14). Within the first 5 years after surgery, recurrence occurs at any site and might involve the liver, the remnant pancreas, lymph nodes, and other sites as lungs and bone, whereas late recurrences seem to affect mainly the liver (14, 17). However, prospective studies based on homogeneous and accurate preoperative diagnostic workup, to avoid stage underestimation at diagnosis, are needed to clarify those findings.

## Clinical and Pathological Risk Factors for Recurrence

### Functioning Status

PanNETs are classified into functioning (F-PanNET) and non-functioning (NF-PanNET) neoplasms according to the presence or the absence of a clinical hormone hypersecretion syndrome. The most common functioning PanNETs are insulinomas, gastrinomas, glucagonomas, and VIPomas. While previously it was suspected that the majority of resected PanNETs were functioning, with insulinomas being the most frequent type, recent data show that between 60 and 90% of PanNETs are non-functional (1, 19). The functioning status has been reported as a favorable characteristic of PanNET, as recurrence occurs in this group in ~4% of cases (9). However, although some F-PanNETs including insulinoma are commonly reported to be benign (20), the prognosis of F-PanNETs is still predominately driven by the tumor stage and pathological features, regardless of hormone secretion (1). It follows that F-PanNETs present with a clinical hormone syndrome that favors diagnosis at early stages. Furthermore, it has been observed that F-PanNETs have a lower median proliferative index and are less likely to have vascular or perineural invasion compared to NF-PanNETs (9). Those features, as discussed later in this review, have a relevant impact on prognosis.

### Symptoms

The presence of hormone clinical syndrome in F-PanNET favors early diagnosis and thus surgical resection at early stages. However, for patients with NF-PanNETs the presence of symptoms at diagnosis is usually related to tumor mass effect or tumoral infiltration on the surrounding structures and therefore is associated with worse prognosis (21, 22). To date, because of the recent increased incidental diagnosis of small NF-PanNETs, fewer patients present with symptoms at diagnosis (23). Abdominal pain is the most frequent symptom occurring in 32–50% of symptomatic patients, while weight loss and jaundice are reported less frequently, respectively, 11–22% and 3–7% of the cases (22–24). Compared to incidental tumors, symptomatic PanNETs present with larger tumor size, higher grade, more frequent lymphovascular, and perineural invasion and are usually detected at an advanced stage (23, 24). Not surprisingly, the presence of symptoms at diagnosis, in patients undergoing surgery (stage I–III), is associated with reduced disease-specific survival and progression-free survival at any stage (23).

### Tumor Grading

Grading of PanNET is based on the proliferation rate of the neoplastic cells, as determined by the mitotic count and/or the Ki67 labeling index. The current 2019 WHO grading system classifies well-differentiated PanNETs into low- (G1), intermediate- (G2), and high-grade (G3) neoplasms (Table 1). Several retrospective studies have validated the prognostic value of PanNET grading, showing that higher grade is associated with an increased risk of recurrence and shorter overall survival, and to date, it is considered the most significant prognostic factor for disease relapse (9, 14, 17, 25, 26). When evaluating the risk of recurrence, G3 PanNETs have a worse prognosis than G1/G2, whereas G2 PanNETs exhibit up to 11-folded risk to recur compared to G1 neoplasms (4, 9). Patients with G3 PanNET need to be strictly surveilled following curative surgery, whereas the outcomes of G1 and G2 neoplasms are more heterogeneous, and their stratification based solely on grade can be inaccurate. G1 and G2 PanNETs represent 95% of all PanNETs undergoing surgical resection (9). Of those, 68–78% are G1 neoplasms. Recurrence is rare in this group and occurs in up to 4% of cases. However, to date, how to discern G1 PanNETs with increased risk of recurring from those that have been definitively cured by surgery is unclear. On the other hand, the category of G2 PanNET is a gray area that includes both indolent and aggressive neoplasms including tumors harboring a Ki67 that widely ranges between 3 and 20% (9). To better stratify patients, several studies have investigated the prognostic role of Ki67, aiming to identify clinically relevant stratification cutoffs. The analysis of large cohorts revealed that in the subgroup of patients with G1 to G2 tumors, the Ki67 cutoff of 5% was the best to stratify prognosis between those two grades (25, 27, 28). In addition, small variations in Ki67 value below 6% cause much larger variations in oncological outcomes, compared to similar variations for higher values of Ki67 (9). Therefore, the actual Ki67 value contributes to predicting prognosis when considered in a continuous, however non-linear, fashion, underscoring the need to develop mathematical tools to interpret Ki67 as a continuous variable.

### Tumor Size

PanNET tumor size has been confirmed as an important prognostic feature. Large tumors are associated with an increased risk to recurrence and worse survival (9, 25). PanNETs larger than 4 cm are, in ≥50% of cases, intermediate-/high-grade neoplasms and with nodal metastases at the time of resection (21). Conversely, NF-PanNETs smaller than 2 cm are usually low-grade tumors (84–95%) with no nodal involvement (86–99%) and uncommonly demonstrate clinical aggressiveness (1, 21, 29). Because of their uncertain malignant potential, the European Neuroendocrine Tumor Society (ENETS) suggests that managing NF-PanNETs ≤2 cm with a “wait and watch” strategy and to limit surgery to those who experience tumor growth during the follow-up. However, 7–17% of small PanNETs have malignant potential based on their tumor grade; therefore, an accurate diagnostic workup must be performed before establishing the best management strategy (21, 30, 31). Small PanNETs undergoing surgery should therefore be followed according to tumor grade, stage, and other pathological features.

### Lymphovascular and Perineural Invasion

Lymphovascular invasion (LVI) is defined as the presence of tumor cells within a definite endothelial-lined lymphatic or blood vessel in the pancreas surrounding the PanNET, while the presence of tumoral cells along nerves or within the layers of nerve fiber is categorized as perineural invasion (PNI). Lymphatic and vascular invasions are usually associated and reported as a single character on the pathology report. Conversely, PNI is a distinct pathologic entity observable in the absence of LVI. The rate of LVI and PNI in PanNETs rages from 22–36% to 17–39%, respectively (9, 14, 22, 32). Vascular and lymphatic vessels and nerves can potentially be a route of metastatic spread to regional lymph nodes and distant organs and are therefore considered a histologic indicator of aggressive tumor behavior. Indeed, the presence of LVI is associated with an x4-8 and PNI x2-6 risk of recurrence (9, 15, 22). Because PanNET recurrence is rare, multivariable analysis of predictive factors is often challenging. As a result, it remains unclear whether LVI and PNI are independent predictors of recurrence. However, although they are associated with larger tumor size and higher tumor grade, they have been often included as separate variables in several prediction tools, suggesting significant contributions in defining prognosis (9, 15, 33).

### Main Pancreatic Duct Involvement

Rarely, PanNET has an infiltrative growth pattern involving the main pancreatic duct (MPD) causing its stenosis or complete obstruction. However, when present it is associated with tumor aggressiveness (34, 35). On imaging, those neoplasms more often arise in the pancreatic head; however, occasionally a clear mass is not visible on imaging and the MPD dilation might be the only suggestive finding (34). Some PanNETs are pathologically characterized by an unusual prominent stromal fibrosis that can involve the MPD, contributing to stenosis and consequent upstream dilation with associated pancreatic atrophy (34). In a series including 101 patients undergoing surgery for PanNET, MPD stenosis has been identified preoperatively, on magnetic resonance cholangiopancreatography images, in 13% of cases and was associated with an increased recurrence rate (50 vs. 7%) (35). These neoplasms are usually larger than 1.5 cm and have frequent nodal involvement (77 vs. 13%) compared to PanNET without MPD involvement, independently of tumor grade (35). Pathologically, strong and diffuse serotonin immunoreactivity has been observed (34).

### Lymph-Node Status

Patients undergoing surgery for PanNET have lymph-node metastasis (pN+) in 26–37% of cases (32, 36, 37). While the association of nodal metastasis with overall survival remains controversial, several studies have now demonstrated the correlate of pN+ with recurrence (6, 9, 16, 32, 36–38). Patients with lymph nodal involvement have a ×5 risk of recurrence following curative resection and a reduced 5-year disease-free survival (DFS) from 86–97% to 60–70% compared to patients with no nodal involvement (36, 37). The ENETS/AJCC staging system classifies PanNET with pN+ as N1, regardless of the nodal burden supported by several studies, suggesting that the number of metastatic lymph nodes fails to impact DFS (36, 37, 39). Several preoperative predictors for pN+ have been identified. On preoperative cross-sectional imaging, the finding of enlarged lymph nodes that might appear hypervascular is strongly suggestive of nodal involvement (36). The use of 68Ga-DOTATATE PET/CT for baseline staging can show a pathological uptake in abdominal retroperitoneal nodal sites with higher accuracy than CT scans (18, 37). Although survival benefit of extended lymphadenectomy has not been proved, formal surgical resection (pancreaticoduodenectomy or distal pancreatectomy) with regional lymphadenectomy should be performed in PanNET at increased risk of nodal disease to allow an accurate pathological staging (6, 19, 36). Lymphadenectomy should always be performed for a PanNET size larger than 4 cm or for those who had a preoperative biopsy showing Ki67 >3%, and gastrinoma due to the high likelihood of having pN+ (19, 36, 37). For those patients at risk of pN+, the optimal number of harvested lymph nodes is 11–15 (6). Finally, during atypical resection such as middle pancreatectomy or enucleation performed to resect selected small NF-PanNETs, nodal sampling may be routinely justified to improve disease staging (40).

### Margin Status

Oncological curative surgery aims to achieve negative resection margins (R0); however, microscopic residual disease on margins (R1) is described in 6–15% of PanNET resections (9, 15, 41). Whether the R1 status is impacting on survival is still debated, and several studies have reported that this condition is associated with recurrence (9, 14, 32, 41, 42). In a previous study, we have observed that patients with R1 margins experienced recurrence in 37 vs. 10% of those with R0 resection (9). Zhang et al. reported a reduced 10-yr recurrence-free survival from 63 to 47% for R1 resections (41). Dong et al., evaluating the pattern of recurrence on a cohort of 1,020 patients, identified the R1 status as an independent prognostic factor for local recurrence but not liver recurrence (14). However, tumors with R1 resection are more likely to be larger, with nodal metastases, LVI, and PNI; it is currently debated whether the margin status is an independently biological metric (14, 41). Finally, the only study evaluating the impact of re-resection of an initially positive margin to achieve R0 demonstrated no benefit in terms of recurrence-free survival or overall survival (41).

### Circulating Biomarkers

#### Serum Chromogranin A

Chromogranin A (CgA) is a glycoprotein stored in the secretory granules of normal neuroendocrine cells and, by measuring in serum or plasma, can be used as a circulating biomarker for the diagnosis and surveillance of PanNETs. Several studies have suggested that CgA is a reliable diagnostic biomarker for PanNETs with increased CgA values associated with higher tumor grade and stage and liver metastasis and might serve as a prognostic marker for both progression-free and overall survival (43, 44). For these reasons, both ENETS and NCCN guidelines advocate serial CgA evaluation during follow-up following curative surgery, whereas NANETS recommends its assessment only for patients with elevated values preoperatively (12, 13, 45). However, increased serum levels are reported in only a quarter of patients with resectable disease and CgA value at diagnosis is not predictive of recurrence after surgery, calling into question CgA clinical utility in this setting (46, 47). Furthermore, CgA increase during follow-up has shown a low positive predictive value, suffering from almost 50% false-positive rates and therefore lacking sufficient specificity to effectively monitor these patients (47, 48). Indeed, CgA levels can increase in association with many other medical conditions such as renal failure and non-neuroendocrine neoplasms, and in patients taking proton-pump inhibitors (46, 49). Finally, interpreting CgA values can be challenging due to the lack of standardization among available assays and measurements across different laboratories, further limiting its use as biomarker for recurrence prediction.

### Peripheral Inflammatory Blood Markers

There is increasing evidence that the systemic inflammatory response plays a role in promoting tumorigenesis and cancer progression for many malignancies (50). The neutrophil–lymphocyte ratio (NLR) is a marker of systemic inflammation which has been reported to predict oncological outcomes in patients with several cancer types (51–54) and can be easily obtained by a routine blood-count analysis. A few retrospective studies have evaluated NLR's role as a biomarker to predict recurrence of PanNET following curative surgery (55, 56). Increased preoperative NLR has been associated with higher Ki67, presence of nodal and liver metastasis, LVI, and PNI (56). Values above 3.4–3.7 at surgery have been found prognostic of recurrence following curative resection; however, NLR values are affected by several other medical conditions as concomitant infection, inflammatory disorders, and use of drugs, including steroids, therefore accurate studies controlling for these factors are required (55, 56). To date, only a small study including 34 patients has prospectively evaluated the prognostic values of NLR for PanNETs undergoing surgery without finding any prognostic relevance (57). Other inflammatory markers were found to be prognostic such as the lymphocyte-to-monocyte ratios and the platelet-to-lymphocyte ratio. However, due to the limited data available, to date, the prognostic significance of these markers needs to be further investigated in larger prospective studies.

### Neuroendocrine mRNA Genomic Biomarker (NETest)

Developing molecular biomarkers detectable by blood-based assays has held great promise to finally facilitate real-time management of the disease for PanNET. NETest is a multi-analyte transcript-based biomarker evaluated on blood samples, extensively investigated over the last few years (58). This test is based upon quantitative reverse-transcription PCR measurement of 51 gene-circulating markers, originally identified by comparing upregulated gastroenteropancreatic neuroendocrine neoplasm (GEP-NEN) transcriptomes and circulating blood transcripts (mRNA) (58, 59). NETest provides a final score ranging between 0 and 100%; a score >20% is diagnostic of neuroendocrine neoplasms (accuracy 95%, specificity 95–98%, sensitivity 89–94%) (59). Changes in NETest levels have been shown to provide meaningful information on the response to treatment with somatostatin analogs and PRRT (59–61). Two prospective studies have also demonstrated that surgical resection of GEP-NEN and PanNET decreases NETest postoperative blood levels and that patients with residual disease have higher levels compared to those receiving an R0 resection (62, 63). Partelli et al. reported that blood transcript levels return to normal (<20%) by 30th postoperative day in 15/30 of patients (63). Among those with persistently high levels, 3 patients had transcript levels >40%, 2 of those with proven residual disease. The remaining 12 patients exhibited only moderate transcript levels (20–40%) in the absence of radiologically detectable disease. Currently, without data on surveillance, the prognostic significance of NETest in this range of values remains unclear. Another study by Genç et al. demonstrated that a NETest value >20% is not uncommon at follow-up of patients with no recurrence following surgery, whereas a cutoff of 40% has an accuracy of 83% in detecting recurrent disease (48). Although results from these preliminary studies are promising, long-term data from these series and further independent prospective studies are still needed to clarify the role of NETest as a biomarker for both detection of residual disease and monitoring patients for recurrence following surgery.

### Prediction Tools

As discussed in this review, there are many clinical and pathological factors associated with recurrence of PanNETs. However, to date, none of them in isolation provides an accurate assessment of recurrence risk for patients undergoing curative surgery of localized disease. The ENETS/AJCC staging system includes tumor size, local disease extent, presence of lymph-node metastases, and distant metastases (TNM system); however, it fails to incorporate tumor-grade assessment, resulting in patients with a different tumor biology included in the same class of risk (9, 39, 64). To overcome this problem and improve prognostication, predictive models and nomograms incorporating multiple variables have been developed, and their characteristics are summarized in Table 2.

**Table 2:** Summary of predictive tools.

	**Reference**					
	**Merath (65)**	**Pulvirenti (9)**	**Genç (15)**	**Zaidi (22)**	**Sho (66)**	**Zou (67)**
Predictive tool type	Nomogram	Nomogram	Scoring system	Scoring system	Scoring system	Scoring system
Study population						
Primary	GEPNEN	Pancreas	Pancreas	Pancreas	Pancreas	Pancreas
Grade	1, 2, 3	1, 2	1, 2	1, 2, 3	1, 2, 3	1, 2
Differentiation	WD, PD	WD	WD	WD, PD	WD, PD	WD
Model cohort n	754	632	211	681	140	245
Model c-index/AUC	0.74	0.85	0.81	n.a.	0.81	0.84
Predictors						
Symptoms	–	–	–	✓	–	–
Tumor diameter	✓	✓	–	✓		
Ki67	✓		–	✓		–
Tumor grade	–	✓		–	✓	
Metastatic lymph node	✓		✓		✓	
Vascular invasion	–	✓	–	–	–	–
Perineural invasion	–	✓		–	–	–
Invasion of adjacent organs	✓	–	–	–	–	–
Validation	Internal independent^*^	External	Internal	Internal independent^**^	Internal	Not validated
Validation cohort n	723	328	–	325	–	–
Validation C-index	0.72	0.84		n.a.		–

* *Pseudo-randomization was used to create two cohorts of patients for the development and validation of the nomogram*;

** *patients were randomized 2:1 to create two cohorts of patients for the development and validation of the score; GEPNEN, gastroenteropancreatic neoplasm; WD, well-differentiated; PD, poorly differentiated; n.a., not available*.

Several studies have developed scoring systems to group patients sharing similar clinicopathological characteristics into defined classes of risk (i.e., low-, intermediate-, and high-risk) (15, 22, 66, 67). In a large study by Zaidi et al. including 1,006 patients, the authors developed and validated a prediction model that assigns points according to the presence of symptoms (1 point), tumor diameter (≥2 cm: 2 points), Ki67 (<3, 3–20, and >20%, respectively, 0, 1, and 6 points), and presence of lymph nodal metastasis (1 point) (22). Based on the final score obtained by summating the points in each category, patients are classified as low-risk (0–2 points), intermediate-risk (3–5 points), and high-risk (6–10 points) of recurrence. Patients in the low-, intermediate-, and high-risk groups had 5, 22, and 56% recurrence rate (*P* < 0.0019). The authors provided a surveillance schedule based on the risk score suggesting a follow-up every 3 months for patients at high risk and every 6 and 12 months respectively for those with an intermediate and low risk to recur. Although this approach is pragmatic and can easily be applied in clinical practice, accuracy remains limited as each category comprises a heterogeneous group of patients. For example, applying this score, patients with G1 or G2 PanNET, >2 cm with pN+, are both classified into the same intermediate risk despite the potential for significantly divergent prognosis. Another scoring system to predict recurrence has been developed by Genc et al. utilizing a cohort of 211 patients. Patients were scored according to tumor grading (G1 and G2, 0 point and 40 points), presence of positive lymph nodes (24 points), and presence of PNI (24 points). While this model potentially allows an estimation of a patient's individual probability of recurrence, only categorical variables were included, limiting the range of possible scores to six categories and with no clear improvements compared to the conventional staging systems (15).

An alternative approach to predict recurrence is represented by nomograms. A nomogram is a graphical representation of mathematical formulas that estimate the individualized risk of a clinical event. This method has recently emerged to be particularly accurate for prognosis prediction in oncology. While in traditional staging systems and risk grouping models continuous variables are converted to categorical, a nomogram allows the incorporation of continuous variable, therefore adding important information provided by the actual value to the model. Compared to risk groups, nomograms are more complex models and their use in clinical practice can be more complicated. However, this increased complexity results in a better predictive accuracy and can be overcome by using electronic versions of nomograms that facilitate the data input, score computing, and risk assessment.

Several groups have proposed this approach, and two different nomograms have been developed to predict PanNET recurrence (9, 22) (Table 2). The US Neuroendocrine Tumor Study Group developed a nomogram on a large cohort of gastroenteropancreatic tumors to predict recurrence following surgery (65). This model includes four variables: Ki67 value, lymph nodal status, tumor size, and presence of invasion of adjacent organs. The model performance was evaluated with a c-index, with 0.71 achieved in the test cohort. This index expresses the ability of the prediction model to distinguish between patients who had recurrence from those who did not. A value of 0.5 indicates that the model is no better than chance, a value above 0.70 identifies a good model, and a value above 0.80 indicates a strong model, whereas a c-index of 1.0 indicates a perfect prediction model (68, 69). Although it was developed on a large cohort of patients who had good performance, this model was not specific for PanNETs, representing a significant limitation as PanNETs have demonstrated different patterns and timescales of recurrence compared with neuroendocrine tumors from other gastrointestinal sites (70). A second nomogram has been proposed by our group, in a collaborative study on a large multi-institutional cohort of surgically resected G1/G2 PanNETs (9). The model has been developed on a cohort of 632 patients treated at two institutions and then externally validated on a cohort of 328 patients undergoing surgery in three different hospitals. The nomogram included four variables: Ki67 value, tumor diameter, number of positive lymph nodes, and presence of LVI and/or PNI. The model obtained promising results as the c-index achieved a value of 0.84 in the validation cohort, which was higher than those achieved by the ENETS/AJCC staging system and WHO grading system (c-index 0.76 for both) and any other prognostic model currently published and validated. Although these results are intriguing, the utility of such tools has not been yet translated into clinical practice. At this time, none of these prognostic models have been prospectively validated nor employed to select patients for clinical trials or to improve surveillance strategies. In addition, none of them have been developed to compute the risk of recurrence after the first 5 years of surgical follow-up.

### Molecular Markers

Over recent years, thanks to the advancements in high-throughput sequencing techniques, the genomic and transcriptomic landscape of sporadic PanNETs has been defined, leading to the identification of recurrent molecular alterations. However, the biological role that each molecular alteration plays in promoting PanNET initiation and progression still requires elucidation. Retrospective genetic studies have shown that some recurrent genetic mutations are associated with an increased risk of metastatic spread, suggesting that their identification might serve as prognostic biomarkers to improve the clinical decision-making process. However, the majority of these findings have not been yet validated in a prospective clinical setting or translated into routine clinical practice.

### Germline Alterations

The initial knowledge of molecular alterations in PanNET was derived from patients with hereditary tumor predisposition syndromes. Familial syndromes are usually caused by a deleterious germline mutation that increases the overall risk of developing a neuroendocrine neoplasm throughout the entire pancreas and in other organs harboring neuroendocrine cells. Key syndromes include multiple endocrine neoplasia type 1 (MEN1), von Hippel–Lindau disease (VHL), neurofibromatosis type 1 (NF1), and tuberous sclerosis complex (TSC), which are characterized by germline mutations in the tumor-suppressor genes *MEN1, VHL, NF1*, and *TSC1* or *TSC2*, respectively.

The MEN1 syndrome is an autosomal-dominant syndrome with a prevalence of 2–3 per 100,000 that affects the pancreas in 30–80% of MEN1 patients, the parathyroid glands, and less frequently the duodenum and the pituitary gland (71). Compared with sporadic PanNET, pancreatic tumors arising in MEN1 patients are characterized by early-onset and multiple pancreatic microadenomas, which can ultimately progress to larger tumors and are often the first neoplastic cause for MEN1 patients' mortality (72, 73). Patients with VHL syndrome present with PanNETs in 10–17% of cases, although other pancreatic neoplasms can be associated with this syndrome, including pancreatic serous cystadenomas and mixed serous cystadenoma-PanNETs (uncommon outside the VHL syndrome) (74). PanNETs are usually well-differentiated, and only occasionally locally advanced or metastatic disease has been reported (75). Pancreatic involvement in NF1 and TSC is less common. In patients with NF1 syndrome, pancreatic tumors are described in 10% of cases; however, these neoplasms are often somatostatinomas that often arise in the duodenum rather than in the pancreas and are characterized by distinct genomic alterations (76, 77). Finally, TSC patients present with pancreatic involvement in only 1%, with both functional and non-functional PanNETs reported (78). Recently, other germline mutations have been described as being associated with PanNET outside these well-known familiar syndromes. Whole-genome sequencing analysis of a large cohort of 98 cases of apparently sporadic PanNETs have identified a higher than expected rate of germline alterations (79). These included germline mutations in MUTYH, whose biallelic inactivation was associated with a novel signature in 5% cases and BRCA2 in 1 case (associated with the respective signature). Germline mutations coupled with LOH were also reported in CHEK2, MEN1, VHL, and CDKN1B (MEN4 syndrome), respectively, in 4, 6, 1, and 1 cases.

### Somatic Mutation

#### MEN 1

*MEN1* mutation is detected in 25–44% of resected tumors while the *MEN 1* locus, on chromosome 11q13, is also frequently lost by chromosomal alterations in 70% of the cases (79–81). The protein-encoded menin is involved in several cellular pathways, including chromatin remodeling, DNA replication, and histone methylation, and *MEN1* mutation has been also correlated with increased telomere length suggesting a role in chromosome maintenance (79). However, MEN1 mutations are independent from those in DAXX and ATRX, which are associated with increased telomere length, indicating that they function in different pathways. Despite the high prevalence of MEN1 mutations, inconsistent results have emerged regarding their potential clinical role. Initial observations on metastatic PanNETs suggested that MEN1 mutations, in combination with DAXX or ATRX mutations, are associated with prolonged survival (80, 82). However, clinical series that specifically investigated the clinical significance of MEN1 loss of function in primary resected PanNET failed to demonstrate a correlation with oncological outcomes (83, 84).

#### mTOR

The mTOR pathway plays a key role in several neoplasms, including PanNETs. Mutations in genes encoding proteins functioning in the mTOR pathway are present in almost 12–15% of PanNETs and include *PTEN, TSC1, TSC2*, and *PIK3CA* and the recently described *DEPDC5* (80). However, besides somatic mutations, other biological mechanisms are involved in the upregulation of the mTOR pathway, as demonstrated by the reduced expression of tumor suppressors functioning in the mTOR axis and the clinical efficacy of agents targeting the pathway, such as everolimus (85, 86). Also, PanNETs harboring mutations in the mTOR pathway have a higher Ki67 and a poor prognosis, suggesting that mutations in these genes might serve as prognostic markers, in particular in the heterogeneous category of G2 tumors (87).

#### DAXX/ATRX

Inactivating somatic mutations in either *DAXX* (25%) or *ATRX* (18%) genes are present in almost half of PanNETs (80). Mutations in *DAXX* or *ATRX* are strongly associated with increased telomere length and are mutually exclusive, confirming that the protein encoded works within the same pathway (88, 89). An increase in telomere length characterizes the alternative lengthening of telomeres (ALT) phenotype, a telomerase-independent mechanism of telomere maintenance, important for the survival of telomerase-negative cancer cells and that has been associated with specific patterns of chromosome alterations (79, 82). The ALT phenotype can be detected on biopsy or resected specimens through telomere-specific FISH and correlate almost perfectly with the DAXX/ATRX status (mutation or protein loss at IHC analysis), whereas only in very rare cases ALT + PanNETs lack mutations in *DAXX* and *ATRX* (88, 90). Initial reports suggested that the ALT phenotype was associated with longer survival in patients with metastatic PanNETs, whereas subsequent studies that specifically investigated ALT prevalence in a large cohort of primary resected PanNETs have shown that ALT, in localized disease, is strongly associated with larger size and higher Ki67 and with metastatic progression (89, 91, 92).

### Gene Expression Signatures

Recent RNA-seq analysis has identified PanNET gene expression signatures that represent distinct endocrine cell lineages and that can predict outcomes following resection (93, 94). The different signatures present similarities with genes that are specifically expressed in islet α- and β-cells and can be specified by the enhanced expression of the transcription factor ARX and PDX1, respectively (94–96). PanNETs with “alpha cell-like” expression form a distinct subgroup that often contain mutation in MEN1, DAXX, or ATRX and an ALT positive phenotype. These tumors are characterized by ARX positivity through IHC and by worse prognosis following resection, especially when associated with ALT (94, 95). PanNETs exhibiting beta-cell lineage-specific gene stain positive for PDX1 infrequently exhibit ALT and rarely recur following resection (94, 95). IHC for ARX and PDX1 are promising factors to assess prognosis; however, further validation on larger cohorts is warranted before they can be considered for clinical application.

## Conclusion

Clinical and pathological factors determining PanNET recurrence after surgery are numerous (Table 3). None of them alone allow an accurate estimation of the risk of recurrence, and it remains unclear which patients should be surveilled closely, with which schedule, and for how long after curative pancreatic resection. Currently, nomograms represent the most accurate and discriminating tools for predicting recurrence in patients with PanNET, enabling the integration of multiple variables. These tools can be used by physicians to provide treatment and follow-up recommendations; however, prospective validation of such models is still required. Moreover, as yet none of these models is capable to of predicting long-term recurrence-free survival (up to 10 year). Therefore, although they can provide help in planning an appropriate follow-up, none is currently capable of selecting of patients for which the postsurgical surveillance can be discontinued. In addition, while many genomic alterations have shown to carry a prognostic significance in retrospective studies, these have not been integrated with clinical and pathological variables in a prospective setting. For future strategies, current clinical prediction tools should be integrated with the results of genomic and transcriptomic sequencing techniques and ALT evaluation. Novel biomarkers, larger data sets, longer follow-up, and more sophisticated modeling procedures will ultimately improve prognostic accuracy and enhance management of this heterogeneous group of neoplasms.

**Table 3:** Summary of most relevant clinical, pathological, and molecular worrisome features for postsurgical recurrence.

**Feature**	**Recurrence risk**	**Clinical significance**	**References**
**Clinical**			
• Functioning status	↓	- Symptoms of clinical hormone syndrome favors the diagnosis at early stages of disease - Commonly low-grade tumor	(1, 9)
• Symptoms in NF-PanNET	↑	- Related to tumor mass effect (large size) and/or tumoral infiltration on the surrounding structures (advanced stage of disease)	(21–24)
**Pathological**			
• Tumor grade	↑	- The most significant prognostic factor for disease relapse - Risk of recurrence increased from G1 to G2 and to G3 neoplasms - The Ki67 value contributes to differentiate prognosis among G2 neoplasms	(4, 9, 14, 17, 25, 26)
• Tumor diameter	↓	- Tumors <2 cm are usually low-grade tumors with no nodal involvement	(1, 21, 29)
	↑	- Tumors >3–4 cm are associated with higher tumor grade and the presence of metastatic lymph nodes	
• Metastatic lymph node	↑	- Associated with ×5 risk of recurrence following curative resection and reduced 5-year DFS	(36, 37)
• Lymphovascular and perineural invasion	↑	- Vascular and lymphatic vessels and nerves can potentially be a route of metastatic spread-Associated with larger tumors and higher tumor grade	(9, 15, 22, 33)
• Main pancreatic duct infiltration	↑	- Caused by tumor-infiltrative growth pattern involving the MPD - Associated with larger tumors and with the presence of nodal metastases	(34, 35)
**Molecular**			
• ALT phenotype	↑	- Associated with larger size and higher Ki67 and with metastatic progression	(79, 89, 91, 92)
• mTOR	↑	- Associated with higher Ki67 and reduced survival in G2 neoplasms	(87)

## Author Contributions

APu and APe wrote and edited the manuscript, created the figure, and created the tables. NJ and DC edited and critically revised the manuscript. All authors read and approved the final manuscript for publication.

## Conflict of Interest

The authors declare that the research was conducted in the absence of any commercial or financial relationships that could be construed as a potential conflict of interest.
